# The P4' Peptide-Carrying *Bacillus subtilis* in Cottonseed Meal Improves the Chinese Mitten Crab *Eriocheir sinensis* Innate Immunity, Redox Defense, and Growth Performance

**DOI:** 10.1155/2024/3147505

**Published:** 2024-02-12

**Authors:** Chao-Fan He, Wei Xiong, Xiang-Fei Li, Guang-Zhen Jiang, Ling Zhang, Zi-Shang Liu, Wen-Bin Liu

**Affiliations:** Key Laboratory of Aquatic Nutrition and Feed Science of Jiangsu Province, College of Animal Science and Technology, Nanjing Agricultural University, No.1 Weigang Road, Nanjing 210095, Jiangsu Province, China

## Abstract

This study developed a recombinant *Bacillus subtilis* to carry the LGSPDVIVIR peptide (cmP4) isolated from the hydrolyzed products of cottonseed meal with excellent antioxidant and immune-enhancing properties in vitro. It was carried as a tandem of five cmP4 peptides (cmP4') to be stably expressed on a large scale. Then, its effectiveness was evaluated in Chinese mitten crab (*Eriocheir sinensis*) based on growth performance, redox defense, and innate immunity. A total of 280 crabs (mean body weight: 41.40 ± 0.14) were randomly assigned to seven diets including a control one (without *B. subtilis*) and six experimental ones with different doses (10^7^,10^8^, and 10^9^ CFU/kg) of unmodified and recombinant *B. subtilis*, respectively, for 12 weeks. Each diet was tested in four tanks of crabs (10/tank). In terms of bacterial dosages, the final weight (FW), weight gain (WG), hemolymph and hepatopancreatic activities of superoxide dismutase (SOD), catalase (CAT), lysosome (LZM), acid phosphatase (ACP) and alkaline phosphatase (AKP), and hepatopancreatic transcriptions of *cat*, mitochondrial manganese superoxide dismutase (*mtmnsod*), thioredoxin-1 (*trx1*), and prophenoloxidase (*propo*) all increased significantly with increasing *B. subtilis* dosages, while hemolymph and hepatopancreatic malondialdehyde (MDA) content and the transcriptions of toll like receptors (*tlrs*), NF-*κ*B-like transcription factor (*relish*), and lipopolysaccharide-induced TNF-*α* factor (*litaf*) all decreased remarkably. In terms of bacterial species, the recombinant *B. subtilis* group obtained significantly high values of FW, WG, hemolymph, and hepatopancreatic activities of SOD, CAT, LZM, ACP, and AKP, and the transcriptions of *mtmnsod*, peroxiredoxin 6 (*prx6*), and *propo* compared with the unmodified *B. subtilis*, while opposite results were noted in hemolymph and hepatopancreatic MDA content and the transcriptions of *tlrs*, *relish*, and *litaf*. These results indicated that dietary supplementation with 10^9^ CFU/kg of recombinant *B. subtilis* can improve the growth performance, redox defense, and nonspecific immunity of *E. sinensis*.

## 1. Introduction

Over the last few decades, aquaculture has become the fastest growing sector of animal food for human consumption. As the world's population continues to grow, a large-scale and intensive culture mode is not only necessary to upgrade the aquaculture industry, but also to feed the increasing population [1, 2]. This culture mode guarantees a high production of aquatic products in a limited space, as boosts the economic efficiency. However, aquatic animals are inevitably subjected to a high-stocking density, and often suffer from health problems due to the reduced antioxidant capacity and compromised immunity. Currently, the increase in aquaculture production from high-stocking densities parallels the increase in the number of disease outbreaks [3, 4]. The industry-wide economic losses due to aquatic animal diseases exceed 6-billion dollars annually [5]. In crustacean aquaculture (e.g., shrimp), losses due to diseases even exceed 40% of the total value of production [6]. This negatively affects aquatic animal welfare, aquaculture production, and economic efficiency, and does not satisfy the needs of sustainability transitions of the aquaculture industry [7]. This emphasizes the urgency to promote the health status of aquatic animals.

With the development of feed processing technologies, plant protein hydrolysates have attracted the attentions of aquaculturists due to their merits in growth enhancement, antimicrobial functions, and antioxidant capacities [8]. Previous studies have excavated multiple functions of cottonseed meal protein hydrolysates in aquatic species, including an improved intestinal digestion and absorption function in *Megalobrama amblycephala* [9], and the enhancements in appetite, organic matter accumulation, and immune function in *Eriocheir sinensis* [10, 11]. These benefits have been mainly attributed to the bioactive peptides derived from the fermentation process. However, these peptides are still poorly characterized until now. In a recent study, the peptide sequences of cottonseed meal protein hydrolysates have been assessed by the electrospray ionization–liquid chromatography–tandem mass spectrometry (ESI–LC–MS/MS) method [9]. Accordingly, a bioactive decapeptide (leucine-glycine-serine-proline-aspartate-valine-isoleucine-valine-isoleucine-arginine (LGSPDVIVIR), cotton meal P4 (cmP4)) has been found with excellent antioxidant and immune-enhancing properties in vitro [12]. In order to further validate its effectiveness in vivo, it is necessary to produce this peptide in a large scale. However, the traditional chemical synthesis method is inefficient and expensive, bringing great difficulties in commercializing this peptide. Therefore, finding an effective way to produce it in a large amount is of great significance.

Chinese mitten crab (*E. sinensis*) is the most important freshwater economic crab in China with an average annual production of about 850,000 tons in the last 3 years [13]. Under intensive culture conditions, *E. sinensis* often suffers from various diseases such as molting disorder, hepatopancreatic necrosis syndrome, and enteritis [14–16]. This inevitably results in a high mortally, thus causing a great economic loss. Therefore, it is of great significance to develop effective nutritional interventions to promote its health status. To date, cottonseed meal protein hydrolysates have been demonstrated to be a health stimulator for *E. sinensis*, but the functional mechanisms still remain elusive. Taking this into consideration, this study creatively constructed a recombinant *Bacillus subtilis* to carry the LGSPDVIVIR peptide isolated from the cottonseed meal protein hydrolysates. Then, its health-promoting effects were evaluated in *E. sinensis* in vivo. The results obtained can promote the healthy cultivation of *E. sinensis*, thereby increasing the profit margins.

## 2. Materials and Methods

### 2.1. Preparation of the Recombinant *B. subtilis*

#### 2.1.1. Purpose and Rationale for Constructing the Recombinant *B. subtilis* Carrying cmP4' Peptide

Our laboratory has previously isolated a decapeptide (leucine-glycine-serine-proline-aspartate-valine-isoleucine-valine-isoleucine-arginine, LGSPDVIVIR) with excellent antioxidant and immune-enhancing bioactivities in vitro from cottonseed meal protein hydrolysate using the electrospray ionization–liquid chromatography–tandem mass spectrometry method, and named it as the cmP4 peptide [12]. A large-scale production of this decapeptide is necessary to further validate its effectiveness in vivo, and to facilitate its application in the aquafeed industry. However, it consists of only 10 amino acids with a relatively small molecular weight, and is easily degraded by host proteases. Therefore, finding an effective approach to express it stably is of great significance. The *B. subtilis* expression system with an effective extracellular protein secretion and a high-heat resistance (able to survive the pelleting process at 80–100°C) fits well with our requirement to express the cmP4 peptide in large quantities. Considering these merits, we tandemly linked five cmP4 peptides from the N-terminus to the C-terminus, and introduced it into the *B. subtilis* expression system. Through this, the amino acid sequence of the tandem cmP4 peptide (cmP4' peptide) was obtained. The tandem expression could improve the stability of the cmP4 peptide, and also increase its copy number. Since the cmP4' peptide contains no lysine, only the terminal arginine serves as a cleavage site for trypsin (a highly conserved protein hydrolase). Therefore, it can be cleaved into the target peptide-cmP4 by endogenous trypsin in the intestine, and is thereby absorbed [17–19].

#### 2.1.2. Construction of the Integrated Plasmid PBE-cmP4'

**Figure 1:**
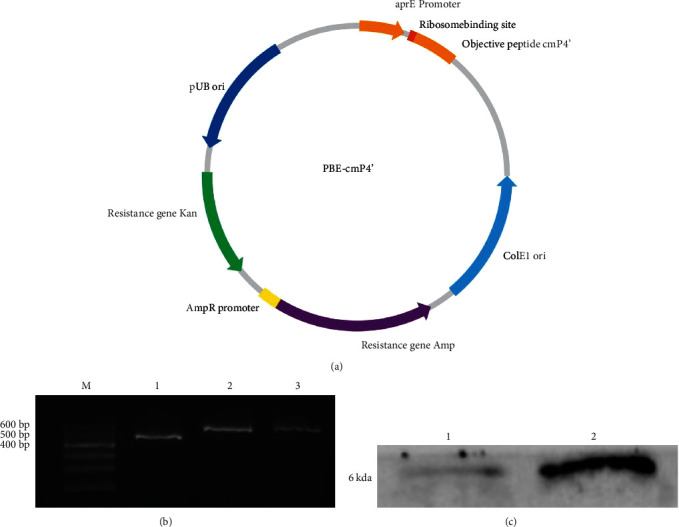
Integrated plasmid PBE-cmP4' (a), PCR identification of positive transformants of recombinant *B. subtilis* (b), and the determined expression of recombinant peptide by western blot (c). In Figure 1(b): M: 600 DNA marker; 1: plasmid without PBE-cmP4'; 2: plasmid with PBE-cmP4'; 3: *B. subtilis* JC66-P4'. In Figure 1(c): 1: secretory expression of cmP4'; 2: inclusion body extraction cmP4'. The results showed that there was a specific band at 6 kda.

Using the genomic DNA of *Escherichia coli* dh5*α* (Jinsirui, Nanjing, China) as template, and F1 (CAAAAAAATGGGTCTACTAA) and R1 (TACAGCATCCAGGGTGACGG) as primers, PCR was conducted to amplify the tandem-cmP4 (cmP4') gene fragment and the cmP4' gene fragment (sequence: CTCGAGATGCATCATCACCACCATCATCGATTGGGTTCTCCGGATGTAATTGTTATCAGACTTGGAAGTCCGGACGTGATTGTTATTAGATTAGGGTCCCCTGACGTTATAGTCATTCGGCTGGGCTCACCTGATGTTATTGTAATCCGTCTCGGCAGCCCAGATGTGATTGTCATCCGC, the underlined part is the homology arm). The *B. subtilis* protease aprE promoter was attached at the upstream of the cmP4' gene fragment with ATG used as the downstream stop codon to constitute the complete cmP4' expression element. The target gene fragment and plasmid PBE (Takara, Dalian, China) were double cleaved by Xhol and SalI restriction endonucleases, respectively. The resulting linear plasmid fragment was ligated by T4 DNA ligase to obtain the integrated plasmid PBE-cmP4' (Figure 1(a)).

#### 2.1.3. Construction of the Recombinant *B. subtilis* JC66-P4'

*B. subtilis* is a Gram-positive bacterium that is cheap and easy to culture, and has a good exocrine capacity in forming spores to secure the survival of the strain under harsh conditions [20], making it a promising carrier of the cmP4 peptide. Accordingly, *B. subtilis* PY79 (NCBI accession number: AHA76664) was activated at 37°C for 12 hr. Then, single colonies of *B. subtilis* PY79 were incubated in the modified Chalmers medium at 37°C for 4 hr. The OD value was measured by a spectrophotometer (Shimadzu, Kyoto, Japan). When the OD value reached 2, 300 *μ*L of *B. subtilis* PY79 was taken out, and was incubated with 30 *μ*L of the integrated plasmid PBE-cmP4' at 37°C for 2 hr. Subsequently, the culture was applied to a plate containing kanamycin, and was incubated upside down at 37°C for 12 hr to obtain *B. subtilis* PY79-cmP4'. Its whole genome was extracted by a commercial kit for Gram-positive bacteria genomic DNA extraction (Qiagen, Hilden, Germany). Then *B. subtilis* JC66 (NCBI accession number: SAMN37990396) was activated following the method as *B. subtilis* PY79. Later, 300 *μ*L of activated *B. subtilis* JC66 was incubated with 30 *μ*L of *B. subtilis* PY79-cmP4' whole genome at 37°C for 2 hr. Then, the culture solution was coated on a kanamycin-containing plate, and was incubated upside down at 37°C for 12 hr to obtain the recombinant *B. subtilis* JC66-cmP4'.

#### 2.1.4. Fermentation Culture of the Recombinant *B. subtilis* JC66-cmP4'

Single colony of the recombinant *B. subtilis* JC66-cmP4' was picked and inoculated in 30 mL of seed medium. After an overnight incubation at 37°C, 180 rpm, the seed solution was inoculated in 50 mL of fresh fermentation medium at 1% inoculum. Then it was incubated again at 37°C, 180 rpm with the fermentation solution sampled and tested every 12 hr. The OD value was 600, the fermentation rate was over 90% at 48 hr, and the budding yield was 6 × 10^9^ CFU/mL.

#### 2.1.5. PCR Identification of the Positive Transformants of Recombinant *B. subtilis* JC66-cmP4'

The recombinant *B. subtilis* JC66-cmP4' obtained was subjected to PCR for rapid validation. The PCR reaction program was: 90°C, pre-denaturation for 30 s; 98°C, denaturation for 10 s; 55°C, annealing for 30 s; 72°C, extension for 1 min, cycling for 30 times, and finally 72°C extension for 2 min. Then, the products were stored at 4°C for subsequent use. The primer sequences for the PCR validation process were: P1: CGAGTCTCTACGGAAATAGC and P2: GCATAACCAAGCCTATGCCTA. The PCR products were detected by an agarose gel electrophoresis with the positive transformants validated (the size is which is 563 bp; Figure 1(b)).

#### 2.1.6. Western Blotting Identification of the Recombinant Peptide

The lysogeny broth medium was used to culture the recombinant *B. subtilis* JC66-cmP4' at 28°C, 200 rpm/min for 240 min. When the OD value of the bacterial solution reached 2, the solution was centrifuged at 4°C, 12,000 rpm for 15 min. The secretion expression cmP4' peptide was collected from the supernatant using the Centricon Plus-70 (Merck Millipore, Germany) centrifugal filtration device (3–10 kda cutoff). The inclusion body extraction cmP4' was collected according to the “Guidebook for Molecule Cloning.” The western blotting assay was performed to identify the secretion expression and the inclusion body extraction of cmP4' peptide. The results showed that there was a specific band at 6 kda (Figure 1(c)).

### 2.2. Experimental Design, Diets, and the Feeding Trial

A 2 × 3 factorial design was adopted in this study, including a control diet (without *B. subtilis*, CON) and six experimental ones incorporating different sources (unmodified and recombinant) and doses (10^7^, 10^8^, and 10^9^ CFU/kg) of *B. subtilis*. The doses of *B. subtilis* were designated according to a previous study [21], which tested the effects of various doses (0, 0.25 × 10^9^, 1 × 10^9^, 2 × 10^9^ and 4 × 10^9^ CFU/kg) of *B. subtilis* on the growth performance of *E. sinensis* with the 1 × 10^9^ CFU/kg group showing the highest weight gain. The diets were abbreviated as BS7, BS8, BS9, RBS7, RBS8, and RBS9, respectively, considering the source (BS: *B. subtilis*, RBS: recombinant *B. subtilis*) and dose of *B. subtilis*. The formulation of the basal diet and the experimental designation were presented in Tables 1 and 2, respectively.

All raw materials were crushed through a 80-mesh sieve. Fish meal, blood meal, soybean meal, cotton meal, peanut meal, rapeseed meal, and *α*-starch were all mixed thoroughly. Then *B. subtilis* was mixed with other feed ingredients in a step-by-step manner, followed by the addition of fish oil and soybean oil. After mixing, distilled water was added at 30% of the raw material mass followed by another thorough mixing. The mixed raw materials were then extruded through a single-screw extruder with a die diameter of 2.0 mm (granulation temperature is 80 ± 5°C, and pressure is 1.6 ± 0.1 kg/cm^2^). The extruded feed was air-dried, cut into 1.8 cm long, and stored at −20°C.

Chinese mitten crabs were provided by the Aquaculture Station of Nanjing Agriculture University (Pukou, Jiangsu province, China). The harvested crabs were kept temporarily in a concrete pond, and fed the basal diet twice daily for 1 week. Two hundred eighty healthy individuals (mean initial body weight: 41.40 ± 0.14 g) were randomly selected and assigned to 28 concrete ponds (0.5 × 0.5 × 0.8 m in length, width, and height) with 10 crabs per pond. Then, each diet was tested in four ponds of crabs. Then, they were subjected to a feeding rate of 4%–6% of body weight for 12 weeks. During this period, the residual feed was cleaned with 1/3 of the water changed daily. The water temperature, dissolved oxygen, pH, and ammonia nitrogen were maintained at 24–28°C, 5 mg/L, 8.0–8.5, and <0.05 mg/L, respectively.

### 2.3. Sample Collection and Calculations

At the end of the culturing period, crabs within each pond were counted and weighed. Two crabs were randomly selected and placed on ice packs for cryoanesthesia. Hemolymph was then obtained from the penultimate pair of paraeiopod using a syringe containing precooling anticoagulant solution [22]. The mixture of hemolymph and anticoagulant solution (1 : 1) was centrifuged at 3,500 rpm for 20 min at 4°C with the supernatant stored at −80°C for subsequent analysis. The hepatopancreas was also dissected on ice packs, and stored at −80°C for subsequent analysis.

The growth-related parameters were calculated as follows:

  Weight gain (WG, %) = (Final body weight (g) − Initial body weight (g) × 100/Initial body weight (g);
  Special growth rate (SGR, %/d) = (ln (Final weight) − ln (Initial weight))/84 × 100;
  Survival rate (SR, %) = 100 × Final survival crab number/Initial crab number;
  Feed conversion ratio (FCR) = dry feed intake (g)/(final body weight(g) − initial body weight (g) + dead crab weight gain (g)).

### 2.4. Analysis of Antioxidant Capability

An appropriate amount of hepatopancreas was weighed and added to a normal saline (*W*/*V*, 1/9) for homogenization at 4°C. Subsequently, the homogenate was centrifuged at 8,500 rpm with the supernatant extracted for enzyme activity analysis. Then, the antioxidant capability was investigated in both hemolymph and hepatopancreas. Hemolymph total superoxide dismutase (SOD) activity was analyzed by the hydroxylamine method [23] at 550 nm. In brief, one unit of SOD is defined as the amount of enzyme per milligram of protein required to produce 50% inhibition of the rate of nitrite production at 37°C. The hepatopancreatic SOD activity was quantified by using the xanthine oxidase method [24] at 550 nm. In brief, one unit of SOD activity was defined as the amount of enzyme required to cause 50% inhibition of xanthine and xanthine oxidase system reaction in 1 mL enzyme extraction of 1 mg protein. The catalase (CAT) activity was measured by measuring the decomposition rate of H_2_O_2_ [25]. The malondialdehyde (MDA) content was determined by the thiobarbituric acid reaction method [26]. In brief, MDA forms a red complex with thiobarbituric acid, having an absorbance at 532 nm. The MDA content is then calculated using the standard curve provided. All the measurements were conducted using commercial kits provided by the Nanjing Jiancheng Bioengineering Institute (Nanjing, Jiangsu, China). SOD, CAT activities, and MDA contents were determined according to commercial kits (No. A001-1-2, A007-1-1, A007-1-1). The OD values were all determined using a UV/visible-6100 Spectrophotometer (Shanghai Precision Instrument Co., Ltd., Shanghai, China).

### 2.5. Measurement of Immune Parameters

The lysozyme (LZM) activity was determined using the turbidimetric method [27]. In brief, the reaction substrate was 0.2 mg/mL of Micrococcus (Sigma) suspension, which was prepared in a 0.05 mol/L, pH 6.1 phosphate buffer. The absorbance was measured at 0.5 and 4.5 min at 530 nm, respectively, with the LZM activity calculated based on the decrease in absorbance per minute of the bacterial solution. Both acid phosphatase (ACP) and alkaline phosphatase (AKP) can decompose disodium benzene phosphate to produce free phenol and phosphoric acid. Phenol interacts with 4-aminoantipyrine in an alkaline solution, and could be oxidized by potassium ferricyanide to produce red quinone derivatives. Through this, the viability of ACP and AKP was determined according to the red coloration at 520 nm. LZM, ACP, and AKP activities were determined according to commercial kits (Nos. A050-1-1, A060-2-1, A059-2-2) provided by the Nanjing Jiancheng Bioengineering Institute (Nanjing, Jiangsu, China), following the instructions provided by the manufacturer, the OD values were all determined using a UV/visible-6100 Spectrophotometer (Shanghai Precision Instrument Co., Ltd., Shanghai, China).

### 2.6. Analysis of Gene Expression

Precisely weigh 100 mg of hepatopancreas in a centrifuge tube, then add 1 mL of RNAiso Plus (Takara, Japan). Insert the centrifuge tube into a beaker filled with dry ice, and homogenize the samples using a handheld homogenizer. After standing for 15 min, add 200 ul of chloroform, and thoroughly mix it. Let it stand for 8 min, then centrifuge (12,000 rpm, 4°C) the mixture for 10 min. The supernatant was mixed with an equal volume of isopropanol, and was left to stand for 8 min. After this, the mixture was centrifuged again, (12,000 rpm, 4°C) for 15 min. The underlayer precipitate was RNA, which was dissolved by mixing with enzyme-free sterile water with its concentration assayed. The diluted RNA was reversely transcribed to cDNA using a reverse transcription kit (Takara, Japan), and was stored at −80°C. The transcriptions of target genes were detected using a SYBR Premix Ex Taq TM II kit with the primers detailed in Table 3. The relative expressions of mRNA were calculated by the 2^−*ΔΔ*CT^ method [37] using the ubiquitin/ribosomal s27 fusion protein (S27) as the house-keeping gene. Base on a previous study, *s27* is the most stable internal reference gene in *E. sinensis* [36]. The gene amplification efficiency was measured, and only primers with an amplification efficiency above 90% were used.

### 2.7. Statistical Analysis

The general linear models in SPSS (IBM SPSS 16.0, SPSS Inc) were used to analyze the significant differences in dietary levels (10^7^, 10^8^, 10^9^ CFU/kg), bacteria species (*B. subtilis* and recombinant *B. subtilis*), and their interactions. Significant differences were determined when the *P* value was less than 0.05. When significance was observed in the interaction, the corresponding data were ranked by the Turkey's multiple range test.

## 3. Result

### 3.1. Growth

As shown in Table 4, there was no significant difference in specific growth rate (SGR), survival rate (SR), and feed conversion ratio (FCR) among the groups (*P* > 0.05). In terms of *B. subtilis* dosage, final body weight (FW) and weight gain (WG) both increased significantly with increasing *B. subtilis* levels (*P* < 0.001). In terms of *B. subtilis* species, significant high values of FW and WG were both noted in the RBS group (*P* < 0.001).

### 3.2. Hemolymph and Hepatopancreas Antioxidant-Related Indicators

As illustrated in Table 5, in terms of *B. subtilis* dosage, the SOD and CAT activities in both hemolymph and hepatopancreas increased significantly with increasing *B. subtilis* levels (*P* < 0.001), whereas the MDA content showed an opposite trend (*P* < 0.001). In terms of *B. subtilis* species, the RBS group showed significant high activities of SOD and CAT compared with the BS group (*P* < 0.001), while an opposite result was noted in the MDA content (*P* < 0.001). In addition, significant interactions between the species and dosages of *B. subtilis* were observed in these indicators except for hemolymph CAT activity and hepatopancreas SOD activity (*P* < 0.01). Furthermore, the highest activities of hemolymph SOD and hepatopancreas CAT, as well as the lowest MDA contents in both tissues were all observed in the RBS9 group.

### 3.3. Hemolymph and Hepatopancreas Immune-Related Indicators

**Figure 2:**
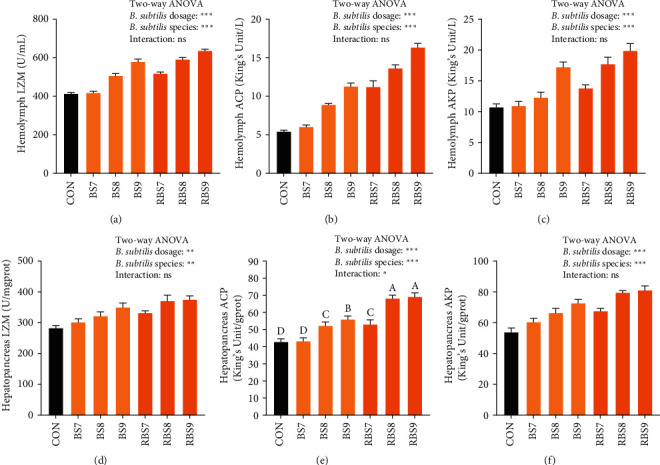
Effects of different dietary levels of *B. subtilis* and recombinant *B. subtilis* on the immunity-related indicators of *E. sinensis*. CON, diet without *B. subtilis*; BS7, dietary supplementation with 10^7^ CFU/kg *B. subtilis*; BS8, dietary supplementation with 10^8^ CFU/kg *B. subtilis*; BS9, dietary supplementation with 10^9^ CFU/kg *B. subtilis*; RBS7, dietary supplementation with 10^7^ CFU/kg recombinant *B. subtilis*; RBS8, dietary supplementation with 10^8^ CFU/kg recombinant *B. subtilis*; RBS9, dietary supplementation with 10^9^ CFU/kg recombinant *B. subtilis*. Hemolymph: lysosome, LZM (a); acid phosphatase, ACP (b); alkaline phosphatase, AKP (c). Hepatopancreas: lysosome, LZM (d); acid phosphatase, ACP (e); alkaline phosphatase, AKP (f). Each data represented the mean of four replicates. Boxes assigned with different superscripts are significantly different (*P* < 0.05). Ns, not significantly different;  ^*∗*^*P* < 0.05;  ^*∗∗*^*P* < 0.01;  ^*∗∗∗*^*P* < 0.001.

As exhibited in Figure 2, in terms of *B. subtilis* dosage, the activities of LZM, ACP, and AKP in both hemolymph and hepatopancreas all increased significantly (*P* < 0.01) with increasing *B. subtilis* levels. In terms of *B. subtilis* species, the RBS group exerted significant high activities of LZM, ACP, and AKP in both hemolymph and hepatopancreas compared with the BS group (*P* < 0.01). In addition, a significant interaction between the species and dosages of *B. subtilis* was observed in hepatopancreas ACP activity (*P* < 0.05) which maximized in the RBS9 group.

### 3.4. Antioxidant and Immune-Related Gene Transcription in the Hepatopancreas

**Figure 3:**
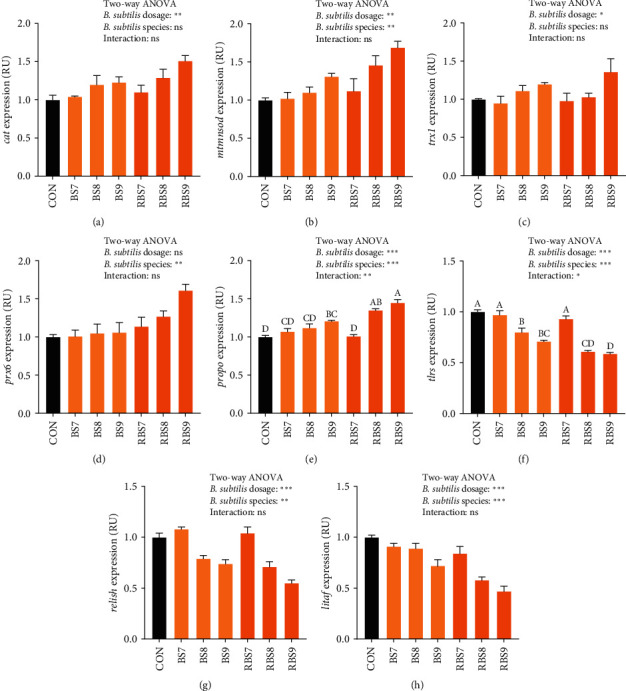
Effects of different dietary dosages of *B. subtilis* and recombinant *B. subtilis* on the transcriptions of catalase (*cat*, a), mitochondrial manganese superoxide dismutase (*mtmnsod*, b), thioredoxin−1 (*trx1*, c), peroxiredoxin 6 (*prx6*, d), prophenoloxidase (*propo*, e), transcriptions of toll like receptors (*tlrs*, f), NF-*κ*B-like transcription factor (*relish*, g), and lipopolysaccharide-induced TNF-*α* factor (*litaf*, h) in the hepatopancreas of *E. sinensis*. CON, diet without *B. subtilis*; BS7, dietary supplementation with 10^7^ CFU/kg *B. subtilis*; BS8, dietary supplementation with 10^8^ CFU/kg *B. subtilis*; BS9, dietary supplementation with 10^9^ CFU/kg *B. subtilis*; RBS7, dietary supplementation with 10^7^ CFU/kg recombinant *B. subtilis*; RBS8, dietary supplementation with 10^8^ CFU/kg recombinant *B. subtilis*; RBS9, dietary supplementation with 10^9^ CFU/kg recombinant *B. subtilis*. For tissue expression, data are referred to the values (relative units (RU)) found in the CON group. Each data represented the mean of four replicates. Boxes assigned with different superscripts are significantly different (*P* < 0.05). Ns, not significantly different;  ^*∗*^*P* < 0.05;  ^*∗∗*^*P* < 0.01;  ^*∗∗∗*^*P* < 0.001.

As presented in Figure 3, in terms of *B. subtilis* dosage, the transcription of peroxiredoxin 6 (*Prx6*) exerted no statistical difference (*P* > 0.05). However, the transcriptions of *cat*, mitochondrial manganese superoxide dismutase (*mtmnsod*), thioredoxin-1 (*trx1*), and prophenoloxidase (*propo*) all increased significantly with increasing *B. subtilis* levels (*P* < 0.05), whereas those of toll like receptors (*tlrs*), NF-*κ*B-like transcription factor (*relish*), and lipopolysaccharide-induced TNF-*α* factor (*litaf*) all showed an opposite result (*P* < 0.001). In terms of *B. subtilis* species, the transcriptions of *cat* and *trx1* both showed no statistical difference (*P* > 0.05). However, the significantly high transcriptions of *mtmnsod, prx6*, and *propo* were all noted in the RBS group compared with the BS group (*P* < 0.01), whereas those of *tlrs, relish*, and *litaf* all showed an opposite result (*P* < 0.05). In addition, significant interactions between the species and dosages of *B. subtilis* were observed in the transcriptions of *propo* and *tlrs* (*P*  < 0.05) with the RBS9 group obtaining the highest transcription of *propo* and the lowest transcription of *tlrs*.

## 4. Discussion

Being a cheap and eco-friendly feed source, plant proteins are a highly potential replacer of fish meal, thereby ensuring sustainable aquaculture. However, their incorporation in aquafeed is highly restricted, due to the existence of antinutritional factors, imbalanced amino acids profiles, poor palatability, low digestibility, and other unknown factors [38]. In addition, under intensive culture, the health of aquatic animals is greatly compromised. Traditionally, antibiotics have been adopted to cope with this issue, resulting in several serious concerns like the development of antibiotic-resistant bacteria, and the antibiotic residues in the aquatic products [39]. Taking them into consideration, this study has constructed a recombinant *B. subtilis* to carry the LGSPDVIVIR peptide (cmP4) isolated from the hydrolyzed products of cottonseed meal with its effectiveness further evaluated on the growth performance and health status of *E*. *sinensis*. The improved growth performance, redox defense, and innate immunity all reinforced the high potential of this peptide as a growth and health-promoter in aquafeed. This suggests that the present technology can largely enhance the nutritional value of the plant proteins, thereby increasing their potentials in substituting fish meal and antibiotics. This could further promote their application in the aquafeed industry, as might ultimately promote sustainable aquaculture.

In the present study, the FW and WG of *E. sinensis* both increased remarkably with increasing dietary *B. subtilis* dosages, suggesting that *B. subtilis* can stimulate the growth of aquatic species. Previous studies have demonstrated that *B. subtilis* can improve the digestive and absorptive capability of aquatic animals by enhancing vitamin synthesis and secreting a wide range of digestive enzymes, thereby improving the feed utilization and growth performance [40–42]. In terms of *B. subtilis* species, relatively high FW and WG were observed in the RBS group compared with the unmodified one, indicating that the recombinant *B. subtilis* has a stronger growth-stimulating effect than the unmodified one. Generally, the cmP4' peptide carried by the RBS can be cleaved by the endogenous trypsin at the arginine site, thereby turning into a single cmP4 peptide [43]. Previously, peptides have been demonstrated to improve the structure of the gastrointestinal tract in aquatic animals, promoting the growth of beneficial bacteria, and increasing the activities of the digestive enzymes, thus exerting the growth-promoting effects [44, 45]. In addition, the cmP4 peptide has been reported to show impressive antioxidant and immune-enhancing effects [46]. It is well known that the growth of animals is closely related to the antioxidant and nonspecific immune capacity [47, 48]. When subjected into oxidative stress, animals often show a compromised growth rate. In addition, if the immunity was inhibited, more nutrients will be allocated to the immune system, as might also negatively affect the growth performance of the aquatic animals [49].

In this study, the activities of hemolymph SOD and CAT of *E. sinensis* both increased significantly with increasing dietary *B. subtilis* dosages. A similar result was also noted in the activities of hepatopancreatic SOD and CAT as well as the transcriptions of *cat, mtmnsod*, and *trx1*, while the opposite was observed in the MDA content. These results indicated that *B. subtilis* can promote the antioxidant capacity of *E. sinensis*. Supportively, both SOD and CAT are important antioxidant enzymes in the body, and the main force to scavenge reactive oxygen species (ROS), thereby resisting oxidative stress [50]. MDA is a lipid peroxidation product, which leads to an increase in cell membrane fragility [51]. In addition, as a cofactor of SOD, *mtmnsod* plays important roles in the ROS scavenging process [52], while *trx1* can attenuate mitochondrial oxidative damage [53]. Furthermore, *prx6* is also a lipid-peroxide–scavenging enzyme, which acts as a cellular protector during oxidative stress [54]. According to a previous study, *B. subtilis* can produce a certain amount of exopolysaccharide, which has excellent anti-inflammatory and antioxidant activities [55]. Similarly, dietary supplementation of the moderate levels of *B. subtilis* also improves the antioxidant capacity of red swamp crayfish (*Procambarus clarkii*) [56] and Chinese mitten crab [57]. In terms of *B. subtilis* species, the RBS group obtained remarkably high activities of SOD and CAT as well as high transcriptions of *mtmnsod* and *prx6*, but a low-MDA content compared with the unmodified one. This suggests that the recombinant *B. subtilis* has a stronger oxidative stress-alleviating effect compared with the unmodified one. This result is in expectation, since peptides are more readily absorbed than whole proteins, and can enhance the nutritional metabolism and also positively regulate the antioxidant defense system of the animals [58, 59]. In addition, excellent antioxidant properties have been observed in the cmP4 peptide in an in vitro study [12]. Notably, an interaction between the dosages and species of *B. subtilis* was noted in hemolymph SOD activity and MDA content as well as hepatopancreatic CAT activity and MDA content with the RBS9 group exerting the best results. This suggests that, when supplied at sufficient dosages, recombinant *B. subtilis* can effectively strengthen the redox defense of *E. sinensis*. Due to the fact that relevant information is barely available, this interaction is hard to explain, thus warranting further studies.

As a crustacean, *E. sinensis* lacks an adaptive immune system. Therefore, the nonspecific immunity is particularly important to maintain its health status. In the present study, the activities of LZM, ACP, and AKP in the hemolymph and hepatopancreas of *E. sinensis* were all significantly elevated with increasing dietary *B. subtilis* dosages. A similar result was also noted in the transcription of *propo* in the hepatopancreas, while those of *tlrs, litaf*, and *relish* all showed an opposite result. The above results indicated that dietary supplementation of *B. subtilis* could improve the nonspecific immunity of *E. sinensis*. Supportively: (1) LZM can exert an antimicrobial effect, and has the ability to stimulate immune response, thereby enhancing the disease resistance of aquatic animals [60]; (2) As a component of phagocytic lysosomes, ACP exerts a bactericidal effect by hydrolyzing phosphate esters on the surface of pathogenic bacteria [61]; (3) AKP is able to label pathogens, which can be the easily recognized by the phagocytoses [62]; (4) The proPO system is considered as an important nonspecific immune system in crustaceans, initiating multiple downstream responses to kill pathogens [63]; (5) Both *tlrs* and *litaf* are important components of the *NF-κB* pathway, and can initiate the immune response [64]; and (6) *relish* participates in the immune response by inducing the expression of antimicrobial peptides [65]. In addition, *tlrs, litaf*, and *relish* are all closely involved in the inflammatory response. An inappropriate activation of *tlrs* leads to the prolonged inflammatory responses [66], while *litaf* can promote the activation of inflammatory cells [67], and *relish* can even directly activate the inflammatory responses in arthropods [68]. Previous studies have shown that *B. subtilis* can directly enhance the immunity of animals by increasing serum immunoglobulin levels [69] and the expression of immune-related genes [70]. Meanwhile, *B. subtilis* also indirectly enhances the immunocompetence by maintaining the intestinal epithelial integrity, downregulating the secretion of inflammatory factors, inhibiting pathogenic bacterial adhesion, and stimulating the growth of immune cells [71, 72]. In terms of *B. subtilis* species, the RBS group obtained remarkably high activities of LZM, ACP, and AKP as well as a high transcription of *propo*, but low transcriptions of of *tlrs, litaf*, and *relish* compared with the unmodified one. This suggests that the recombinant *B. subtilis* has a stronger immune-stimulating effect than the unmodified one. According to a previous study, recombinant *B. subtilis* expressing immunologically active heterologous peptides is effective in stimulating systemic immune responses [73, 74]. In addition, peptides are able to ameliorate intestinal damage and maintain intestinal mucosal integrity [75], thereby maintaining intestinal immune homeostasis. It is greatly acknowledged that the intestinal mucosa is the first line of immune defense [76], and pathogenic microorganisms need to cross the intestinal mucosa to invade the spleen and liver [77]. Notably, an interaction between the dosages and species of *B. subtilis* was observed in hepatopancreas ACP activity and the *propo* and *tlrs* transcripts with the RBS9 group exerting the best results. This suggests that, when supplied at sufficient dosages, recombinant *B. subtilis* can exert immune-enhancing advantages. The potential mechanisms underlying this interaction are still unknown, as needs further in-depth studies.

## 5. Conclusion

This study creatively constructed a recombinant *B. subtilis* to carry the LGSPDVIVIR peptide isolated from cottonseed meal protein hydrolysates with its health-promoting effects evaluated in *E. sinensis*. The results showed that dietary supplementation with 10^9^ CFU/kg of recombinant *B. subtilis* remarkably improved the growth performance, redox defense, and innate immunity of *E. sinensis*. However, the best result was noted in the highest dose group, suggesting that the optimal dietary level of recombinant *B. subtilis* still needs to be investigated in the future studies. Despite this, the present findings could to some extent promote the application of plant proteins in aquafeed, and provide novel antibiotic replacement strategies for the crustaceans.

## Figures and Tables

**Table 1:** Formulation and proximate composition of the experimental basal diets.

Ingredients (%)	
Fish meal	30.00
Blood meal	4.00
Soybean meal (defatted)	10.00
Cottonseed meal	4.00
CPH	0
Peanut meal	18.81
Rapeseed meal	2.00
*α*-Starch	20.93
Soybean oil	3.55
Fish oil	1.00
Ca(H_2_PO_4_)_2_	1.50
Zeolite powder	0.9
Premix^a^	1.00
Mixture^b^	2.30
Proximate composition (% dry-matter basis)	—
Dry matter	89.46
Crude protein	39.78
Crude lipid	7.51

*Note*: CPH, cottonseed meal protein hydrolysate. ^a^Premix supplied the following minerals (g/kg) and vitamins (IU or mg/kg) per kg: CuSO_4_ · 5H_2_O, 2.0 g; FeSO_4_ · 7H_2_O, 25 g; ZnSO_4_ · 7H_2_O, 22 g; MnSO_4_ · 4H_2_O, 7 g; Na_2_SeO_3_, 0.04 g; KI, 0.026 g; CoCl_2_ · 6H_2_O, 0.1 g; Vitamin A, 900,000 IU; Vitamin D, 200,000 IU; Vitamin E, 4,500 mg; Vitamin K_3_, 220 mg; Vitamin B_1_, 320 mg; Vitamin B_2_, 1,090 mg; Vitamin B_5_, 2,000 mg; Vitamin B_6_, 500 mg; Vitamin B_12_, 1.6 mg; Vitamin C, 10,000 mg; Pantothenate, 1,000 mg; Folic acid, 165 mg; Choline, 60,000 mg; Biotin, 100 mg; *Myo*-inositol 15,000 mg. ^b^Mixture includes the following ingredients (%): choline chloride 4.21%; antioxidants 1.26%; mildew-proof agent 2.09%; salt 21.03%; lvkangyuan 63.15% and biostimep 8.26%.

**Table 2:** The designation of experimental treatments.

Groups	Treatments
CON	Basal diet
BS7	Basal diet + 10^7^ CFU/kg *B. subtilis*
BS8	Basal diet + 10^8^ CFU/kg *B. subtilis*
BS9	Basal diet + 10^9^ CFU/kg *B. subtilis*
RBS7	Basal diet + 10^7^ CFU/kg recombinant *B. subtilis*
RBS8	Basal diet + 10^8^ CFU/kg recombinant *B. subtilis*
RBS9	Basal diet + 10^9^ CFU/kg recombinant *B. subtilis*

*Note*: BS, *B. subtilis*; RBS, recombinant *B. subtilis*.

**Table 3:** Nucleotide sequences for real-time PCR primers.

Target genes	Forward (5′−3′)	Reverse (5′−3′)	References
*cat*	ATCAAGTGTCATTCCTCTTCTCTG	CCTTCCCTTCTTTGTTCACCA	[28]
*mtmnsod*	AAGGTTCTGGTTGGGGCT	AACATTCTTGTACTGCAG	[29]
*trx1*	TCGAGACTACATCGCTAAGTACAAA	AAACTCCACTCCGAGCATCC	[30]
*prx6*	ACCCATCGGACTACACCCCAG	GGACCAATGACAAAGACAGCA	[31]
*propo*	CCATCCCTTCCTGCTTACCA	CTCCATCACAAACCCTAACGACTT	[32]
*tlrs*	CTCCTTCACCTGCCCTAACTGCT	CTCCAGTTTGTATTGCTGTGCGAAA	[33]
*relish*	TCTCCCTACTCTGACCATTCC	TTCCCACCATCTCACTCTTGT	[34]
*litaf*	CAGGAGTAGTGTCGGGATTTGC	AGTTGTTGGAGCAGCACCTTG	[35]
*s27*	GGTCGATGACAATGGCAAGA	CCACAGTACTGGCGGTCAAA	[36]

*Note: cat*, Catalase; *mtmnsod*, mitochondrial manganese superoxide dismutase; *trx1*, thioredoxin-1; *prx6*, peroxiredoxin 6; *propo*, prophenoloxidase; *tlrs*, toll like receptors; *relish*, NF-*κ*B-like transcription factor; *litaf*, lipopolysaccharide-induced TNF-*α* factor; *s27*, ubiquitin/ribosomal S27 fusion protein.

**Table 4:** Effects of different dietary levels of *B. subtilis* and recombinant *B. subtilis* on the growth performance of *E. sinensis*.

Groups	IW (g)	FW (g)	WG (%)	SGR (%)	SR (%)	FCR
CON	41.40 ± 0.14	72.90 ± 0.15	76.08 ± 0.27	0.81 ± 0.01	72.50 ± 2.50	2.19 ± 0.05
BS7	41.30 ± 0.10	74.38 ± 0.10	80.08 ± 0.17	0.84 ± 0.01	80.00 ± 4.08	2.03 ± 0.05
BS8	41.60 ± 0.08	75.61 ± 0.08	81.75 ± 0.10	0.86 ± 0.01	77.50 ± 4.79	2.01 ± 0.06
BS9	41.40 ± 0.14	76.31 ± 0.19	84.33 ± 0.43	0.87 ± 0.01	75.00 ± 2.89	2.01 ± 0.03
RBS7	41.35 ± 0.17	75.51 ± 0.25	82.62 ± 0.50	0.86 ± 0.01	82.50 ± 2.50	2.09 ± 0.07
RBS8	41.30 ± 0.19	76.78 ± 0.18	85.89 ± 0.25	0.89 ± 0.01	82.50 ± 2.50	2.05 ± 0.02
RBS9	41.45 ± 0.17	78.13 ± 0.85	88.50 ± 2.09	0.91 ± 0.05	77.50 ± 4.79	1.95 ± 0.13
*B. subtilis* dosage (CFU/kg)						
10^7^	41.33 ± 0.09	74.94 ± 0.27^c^	81.35 ± 0.64^c^	0.85 ± 0.01	81.25 ± 2.64	2.06 ± 0.05
10^8^	41.45 ± 0.11	76.19 ± 0.27^b^	83.82 ± 0.64^b^	0.87 ± 0.01	80.00 ± 2.64	2.03 ± 0.05
10^9^	41.43 ± 0.10	77.22 ± 0.27^a^	86.42 ± 0.64^a^	0.89 ± 0.01	76.25 ± 2.64	1.98 ± 0.05
*B. subtilis* species						
* B. subtilis*	41.43 ± 0.07	75.43 ± 0.22^b^	82.05 ± 0.52^b^	0.86 ± 0.01	77.50 ± 2.15	2.01 ± 0.04
Recombinant *B. subtilis*	41.37 ± 0.09	76.81 ± 0.22^a^	85.67 ± 0.52^a^	0.88 ± 0.01	80.83 ± 2.15	2.03 ± 0.04
Two-way ANOVA						
* B. subtilis* dosage	ns	^*∗∗∗*^	^*∗∗∗*^	ns	ns	ns
* B. subtilis* species	ns	^*∗∗∗*^	^*∗∗∗*^	ns	ns	ns
Interaction	ns	ns	ns	ns	ns	ns

*Note*: Values are means ± SE of four replications. Means in the same column with different superscripts are significantly different (*P* < 0.05). IW, initial body weight; FW, final body weight; WG, weight gain; SGR, special growth rate; SR, survival rate; FCR, feed conversion ratio. Ns, not significantly different;  ^*∗∗∗*^, *P* < 0.001.

**Table 5:** Effects of different dietary levels of *B. subtilis* and recombinant *B. subtilis* on the antioxidant-related indicators of *E. sinensis*.

Groups	Hemolymph			Hepatopancreas		
	SOD (U/mL)	CAT (U/mL)	MDA (nmol/mL)	SOD (U/mgprot)	CAT (U/gprot)	MDA (nmol/mgprot)
CON	303.62 ± 2.97^d^	10.28 ± 0.05	4.41 ± 0.08^a^	59.92 ± 0.60	4.37 ± 0.02^e^	15.40 ± 0.11^a^
BS7	307.97 ± 4.70^cd^	10.57 ± 0.20	4.07 ± 0.06^b^	58.92 ± 0.35	4.31 ± 0.03^e^	12.79 ± 0.03^b^
BS8	321.38 ± 2.52^bc^	10.83 ± 0.06	3.69 ± 0.06^c^	63.93 ± 0.88	4.26 ± 0.04^e^	11.04 ± 0.16^c^
BS9	329.45 ± 4.84^b^	11.24 ± 0.19	3.25 ± 0.07^d^	65.13 ± 0.99	4.61 ± 0.05^d^	8.32 ± 0.11^d^
RBS7	328.62 ± 3.63^b^	11.13 ± 0.16	2.99 ± 0.11^d^	65.75 ± 0.49	4.96 ± 0.07^c^	7.48 ± 0.13^e^
RBS8	367.99 ± 2.87^a^	12.15 ± 0.10	2.28 ± 0.07^e^	68.22 ± 1.09	5.27 ± 0.04^b^	6.54 ± 0.06^f^
RBS9	375.44 ± 3.53^a^	12.25 ± 0.17	2.18 ± 0.04^e^	70.66 ± 0.69	5.70 ± 0.08^a^	6.10 ± 0.06^f^
*B. subtilis* dosage (CFU/kg)						
10^7^	318.30 ± 2.67^c^	10.85 ± 0.11^b^	3.53 ± 0.05^a^	62.34 ± 0.56^c^	4.63 ± 0.04^c^	10.14 ± 0.07^a^
10^8^	344.68 ± 2.67^b^	11.49 ± 0.11^a^	2.94 ± 0.05^b^	66.07 ± 0.56^b^	4.76 ± 0.04^b^	8.79 ± 0.07^b^
10^9^	352.45 ± 2.67^a^	11.74 ± 0.11^a^	2.76 ± 0.05^c^	67.89 ± 0.56^a^	5.15 ± 0.04^a^	7.21 ± 0.07^c^
*B. subtilis* species						
* B. subtilis*	319.60 ± 2.18^b^	10.88 ± 0.09^b^	3.67 ± 0.04^a^	62.66 ± 0.46^b^	4.39 ± 0.03^b^	10.71 ± 0.07^b^
Recombinant *B. subtilis*	357.35 ± 2.18^a^	11.84 ± 0.09^a^	2.48 ± 0.04^b^	68.21 ± 0.46^a^	5.31 ± 0.03^a^	6.70 ± 0.07^a^
Two-way ANOVA						
* B. subtilis* dosage	^*∗∗∗*^	^*∗∗∗*^	^*∗∗∗*^	^*∗∗∗*^	^*∗∗∗*^	^*∗∗∗*^
* B. subtilis* species	^*∗∗∗*^	^*∗∗∗*^	^*∗∗∗*^	^*∗∗∗*^	^*∗∗∗*^	^*∗∗∗*^
Interaction	^*∗∗*^	ns	^*∗∗*^	ns	^*∗∗*^	^*∗∗∗*^

*Note*: Values are means ± SE of four replications. Means in the same column with different superscripts are significantly different (*P* < 0.05). SOD, superoxide dismutase; CAT, catalase; MDA, malondialdehyde. Ns, not significantly different;  ^*∗∗*^, *P* < 0.01;  ^*∗∗∗*^, *P* < 0.001.

## Data Availability

The data of this research can be obtained from the corresponding author under reasonable requirements.
